# MULTISTATE CHARACTERISTICS AMONG OLDER POPULATION IN CHINA (2024–2050): GLOBAL COMPARISON AND DYNAMIC SIMULATION

**DOI:** 10.1093/geroni/igae098.2663

**Published:** 2024-12-31

**Authors:** Yichao Li, Hong Mi

**Affiliations:** Zhejiang University, Hangzhou, Zhejiang, China (People’s Republic); Zhejiang University, Hangzhou, Zhejiang, China (People’s Republic)

## Abstract

Global long-term care (LTC) issues become complex with diverse aging process. China’s population has entered deep and accelerated aging stage with multiple health status, which has heterogeneity among population with different features. However, overlooking the biases of multistate characteristics of aging due to lack of systematic methodology would affect LTC policies. Therefore, this study simulates the complex LTC demand by estimating life expectancy with disability and structure of disability population under different demographic scenarios, and compares China’s LTC demands with typical developed countries. In methods part, we used three-parameter lifetable model (DCMD model), Lee-Carter Coherent model and Bayesian multistate model to simulate the prevalence of mild, moderate and severe disability among older people. China Health and Retirement Longitudinal Study (CHARLS) 2020 was used for the initial dependency status. CHARLS 2011-2018 and Chinese Longitudinal Healthy Longevity Survey 2008-2014 was used as prior information. Death probabilities in Bayesian health transition matrixes were modified by estimated mortality patterns by sex, age and residential types (urban or rural areas). Healthy life expectancy in 2020 was also calculated by multistate lifetable model. Results showed that older people with moderate and severe disability in China had an increasing trend during 2023 to 2050 after simulating posterior distributions of health transition probabilities. China’s public long-term care expenditures will account for less than 0.7% of GDP in 2050, which is lower than developed countries. This study proposed an actuarial framework for estimating LTC demand with credible interval, which enables developing countries to dynamically adjust LTC policies.

